# Injectable and in situ crosslinkable gelatin microribbon hydrogels for stem cell delivery and bone regeneration *in vivo*

**DOI:** 10.7150/thno.41096

**Published:** 2020-05-15

**Authors:** Yaohui Tang, Xinming Tong, Bogdan Conrad, Fan Yang

**Affiliations:** 1 Department of Orthopaedic Surgery, Stanford University School of Medicine, Stanford, CA, 94305, USA.; 2 Program of Stem Cell Biology and Regenerative Medicine, Stanford University School of Medicine, 300 Pasteur Dr., Edwards R105, Stanford, CA, 94305, USA.; 3 Department of Orthopaedic Surgery, Stanford University School of Medicine, 300 Pasteur Dr., Edwards R105, Stanford, CA, 94305, USA.

**Keywords:** bone, hydrogels, injectable, macroporous, stem cells

## Abstract

**Rationale**: Injectable matrices are highly desirable for stem cell delivery. Previous research has highlighted the benefit of scaffold macroporosity in enhancing stem cell survival and bone regeneration in vivo. However, there remains a lack of injectable and in situ crosslinkable macroporous matrices for stem cell delivery to achieve fast bone regeneration in immunocompetent animal model. The goal of this study is to develop an injectable gelatin-based μRB hydrogel supporting direct cell encapsulation that is available in clinics as macroporous matrices to enhance adipose-derived stromal cell (ASC) survival, engraftment and accelerate bone formation in craniofacial defect mouse.

**Methods**: Injectable and in situ crosslinkable gelatin microribbon (μRB)-based macroporous hydrogels were developed by wet-spinning. Injectability was optimized by varying concentration of glutaraldehyde for intracrosslinking of μRB shape, and fibrinogen coating. The efficacy of injectable μRBs to support ASCs delivery and bone regeneration were further assessed in vivo using an immunocompetent mouse cranial defect model. ASCs survival was evaluated by bioluminescent imaging and bone regeneration was assessed by micro-CT. The degradation and biocompatibility were determined by histological analysis.

**Results**: We first optimized injectability by varying concentration of glutaraldehyde used to fix gelatin μRBs. The injectable μRB formulation were subsequently coated with fibrinogen, which allows in situ crosslinking by thrombin. Fluorescence imaging and histology showed majority of μRBs degraded by the end of 3 weeks. Injectable μRBs supported comparable level of ASC proliferation and bone regeneration as implantable prefabricated μRB controls. Adding low dosage of BMP2 (100 ng per scaffold) with ASCs substantially accelerated the speed of mineralized bone regeneration, with 90% of the bone defect refilled by week 8. Immunostaining showed M1 (pro-inflammatory) macrophages were recruited to the defect at day 3, and was replaced by M2 (anti-inflammatory) macrophages by week 2. Adding μRBs or BMP2 did not alter macrophage response. Injectable µRBs supported vascularization, and BMP-2 further enhanced vascularization.

**Conclusions**: Our results demonstrated that µRB-based scaffolds enhanced ASC survival and accelerated bone regeneration after injection into critical sized cranial defect mouse. Such injectable µRB-based scaffold can provide a versatile biomaterial for delivering various stem cell types and enhancing tissue regeneration.

## Introduction

Stem cell transplantation has shown great potential for tissue regeneration. However, delivering cells alone usually results in poor cell survival and engraftment 1-3, and do not provide any structural support 4. To enhance the therapeutic outcomes, biomaterials have been widely used as three-dimensional artificial niche for cell delivery with tunable biochemical and physical cues 5-7. Hydrogels are popular biomaterials for cell delivery due to injectability, ease of cell encapsulation, and tissue-like water content 8, 9. However, hydrogels are crosslinked polymeric network, which typically contain mesh size orders of magnitude smaller than the size of cells 10. To overcome such physical constraints, degradation may be introduced to support cell proliferation and new matrix deposition, but the ability of cells to degrade matrices vary and degradation takes time, often leading to undesirable delayed tissue regeneration 11.

In addition to degradation, macroporosity have also been introduced to scaffolds to help overcome physical constraint and accelerate the speed of vascularization and new tissue regeneration 12-14. Conventional methods to introduce macroporosity include particle leaching 15, phase separation 16, gas foaming 17, micro-extrusion 18, electrospinning 19, and 3D printing 20. However, these methods often involve steps that are not cell-friendly, and cells can only be seeded onto the prefabricated scaffolds. This results in undesirable heterogeneous cell distributions and do not support injectability and in situ polymerization.

Given the limitations of conventional macroporous scaffolds, injectable scaffolds have become more attractive due to the ease of handling and reduced invasiveness 21. However, the injectable scaffolds with macroporosity are limited. Approaches have been exploited to fabricate injectable macroporous scaffolds for cell delivery 13, 22. For example, macroporous cryo-hydrogels have been developed as cell carrier, which possess shape-memory properties that withstand reversible deformation and allow rapid volume recovery post injection 23-25. These cryo-hydrogels have shown to support cell retention and survival post-injection in vivo. However, in order to allow homogenous cell infiltration and easier injection, these cryo-hydrogels can only be used with relatively small size, ranging from hundreds of micrometers to a few millimeters. In addition, these porous scaffolds are prefabricated, which could limit cell infiltration by the pore size and the loading method.

In contrast, the bottom-up approaches using injectable building blocks to build up macroporous scaffolds in situ are more facile and advantageous, due to the ensured interconnected macroporosity and homogeneous cell distribution. For example, injectable hydrogel microspheres can be annealed to form macroporous scaffolds 26, 27. These microparticles were packed in high density to form a 3D scaffold, with pores formed by empty spaces among the annealed spheres. The interconnected macroporosity substantially enhanced cell proliferation and helped integrating newly formed tissue with host tissue in the wound-healing model 26, 27. However, this strategy requires high packing density to ensure contact between the microspheres, which could limit available porosity. The low ratios of surface area to volume may result in limited surface area to support cell seeding and growth. In addition, the relatively weak mechanical strength (10-1000 Pa) could limit its application for treating load-bearing tissues 26.

Compared to microspheres, building blocks with the geometry of fibers and ribbons can facilitate the inter-connection and provide higher surface area and porosity. For example, our lab has recently reported the development of a gelatin-based scaffold using microribbon (μRB)-shaped hydrogels as building blocks that combines injectability and macroporosity 28. The μRB-based scaffold allows direct cell encapsulation in macroporous niche with homogenous cell distribution and robust proliferation 28. In addition, the μRBs has high diameter-to-length aspect ratio and high inter-connectivity in the scaffolds. These features render the µRB scaffolds unique high mechanical flexibility and resilience, which can recover the original shape after 90% cyclic-strain compression 28, 29. This makes it appropriate for engineering load-bearing tissues such as bone and cartilage. Delivering adipose-derived stromal cells (ASCs) using the μRB-based macroporous scaffolds resulted in significantly increased cell survival in vivo and enhanced bone healing as compared to conventional nanoporous hydrogels 30.

While our previous work showed the potential of gelatin µRBs as macroporous matrices for enhancing stem cell survival in vivo, several key bottlenecks remain before the translational potential of gelatin µRBs for cell delivery can be fully realized. First, our previous gelatin µRBs were prefabricated in vitro and not yet optimized for injection. Previous studies have shown cell viability are often compromised during injection due to increased shear forces, and specific optimization of the µRB properties must be performed to identify optimized µRB formulation that supports high cell viability after injection. Second, our previous gelatin µRBs require light for polymerization, which are not suitable for polymerization in deep tissues with poor light penetration. Third, the speed of mineralized bone formation using gelatin µRBs and ASCs remain slow and unsatisfactory, and degradation and biocompatibility of gelatin µRBs in vivo remains largely unknown. To overcome these limitations, the goal of this study is to develop injectable gelatin-based µRBs that can form macroporous scaffolds in situ to support stem cell survival and bone regeneration in an immunocompetent mouse cranial defect model. We first assessed the degradation and inflammatory responses of optimized injectable gelatin-based µRB scaffolds in vivo. We further exploited the potential of using the µRBs to deliver BMP-2 and examined the synergy of BMP-2 with ASCs to promote bone regeneration.

## Materials and Methods

### Fabrication of gelatin-based microribbons (μRBs)

Gelatin-based μRBs were fabricated by wet-spinning as we previously reported 28. Briefly, type-A gelatin (Sigma, USA) was dissolved in dimethyl sulfoxide (17% wt/wt) at 60 °C overnight, and ejected at 5 ml/h at room temperature into ethanol bath with constant stirring. The collected microfibers were transferred into acetone, with solvent exchange causing asymmetrical collapse of the microfibers into the microribbon (µRB) shape. To lock the μRB shape, the gelatin µRBs were first intra-crosslinked in glutaraldehyde (GTA) solution under stirring overnight, neutralized for 2 h in L-lysine hydrochloride (1%) to block unreacted aldehyde groups, and washed at least six times with deionized water. To allow inter-crosslinking among gelatin μRBs later for cell encapsulation, fixed μRBs were suspended in fibrinogen (FB) solution (0.1% or 0.5%) overnight, and then washed eight times with deionized water. The µRB products were then freeze-dried and stored at -20°C before use. For in vivo tracking of degradation of μRBs, gelatin μRBs were labeled with Alex floure-700 or rhodamine. Specifically, 1 mg of Alexa Fluor 700 NHS Ester (Thermo Scientific, Barrington, IL, USA) or rhodamine NHS Ester (Thermo Scientific) was added into 200 ml glutaraldehyde solution for labeling 1 g μRBs.

### Characterizing the stiffness of μRBs using atomic force microscopy

The stiffness of individual gelatin μRB is tuned by varying concentration of glutaraldehyde (12.5%, 20% and 37.5%), which controls the degree of polymerization between amine groups within gelatin and glutaraldehyde. The stiffness of the µRBs was measured in PBS at 37°C using a NX-10 atomic force microscope (Park System), equipped with a probe with silicon nitride cantilever with 2 μm colloidal tip (NanoAndMore). A non-contact mode with indenting speed at 1 µm/s was used, and the stiffness was calculated by fitting the loading portion of each force-distance curve to the Hertzian model, assuming a Poisson's ratio of 0.4 for the samples. A total of 4-6 areas were measured in each μRB sample, and 5 μRB samples per group were tested.

### Characterizing inter-crosslinked macroporous μRB scaffolds using scanning electron microscopy

The morphology of inter-crosslinked gelatin μRB scaffolds was assessed using a Hitachi S-3400N variable pressure scanning electron microscope (VP-SEM). Samples were incubated in PBS at 37 °C overnight and rinsed with DI water before being loaded to the chamber of SEM. The hydrated samples were gradually cooled from room temperature to -25 °C as the chamber pressure reduced from 1 atm to 50 Pa, following a P/T curve at which water stays liquid phase. The samples were imaged under the electron beam intensity at 15 kV and a working distance around 7 mm.

### Isolation, characterization and differentiation of adipose-derived stromal cells (ASCs)

All procedures involving animals were approved by the Institutional Animal Care and Use Committee of Stanford University. Mouse ASCs were isolated from inguinal fat pads of 8-week-old, luciferase-positive transgenic mice (Jackson labs) 30. Briefly, fat tissues were washed with HBSS and digested with Liberase enzymes (Roche Diagnostics, Indianapolis, IN) at 37°C for 1h. Then enzyme activity was neutralized with fetal bovine serum (FBS, Invitrogen, Carlsbad, CA) and cells were filtered through a 40-μm cell strainer to remove cellular debris, and seeded into 75 cm^2^ flasks. Following cultured at 37°C with 5% CO_2_ in an incubator (Thermo Scientific, Barrington, IL, USA) for 2 days, cells were washed with PBS and expanded in Dulbecco's Modified Eagle Medium (DMEM) with fetal bovine serum (FBS, 10%), penicillin/streptomycin (100 U/ml). Cells were passaged upon 85-90% confluence and second-passage cells were used for all experiments.

ASCs were characterized by flow cytometry (FACS Arial II; BD Falcon, USA) using anti-CD73 (1:100; eBioscience, San Diego, CA, USA), anti-CD90 (1:100; eBioscience), anti-CD105 (1:100; eBioscience) and anti-CD45 (1:100; eBioscience) antibody markers, according to the manufacturer's instruction.

The in vitro differentiation capacity of the isolated ASCs was characterized. To induce osteogenic differentiation of ASCs, cells were cultured in osteogenic medium containing DMEM with FBS (10%), beta glycerol phosphate disodium salt (10 mM), dexamethasone (100 nM), L-ascorbic acid 2-phosphate (A2P, 50 μg/ml) and penicillin/streptomycin (100 U/ml). For adipogenic induction, ASCs were incubated with adipogenic medium which was comprised of DMEM, FBS (10%), penicillin/streptomycin (100 U/mL), dexamethasone (1 μM, Sigma), indomethacin (10 μM, Sigma), 3-isobutyl-1-methylxanthine (0.5 mM, Sigma), and insulin (10 μg/mL, Sigma). Medium was changed every 2-3 days. Cells were fixed with 4% paraformaldehyde and stained with alkaline phosphatase kit and Alizarin Red S (for osteogenesis) and oil red (for adipogenesis) according to the manufacturer's instructions (STEMPRO, Gibco).

### Validation of injectability and in situ crosslinkability of μRB scaffolds

To test injectability of μRBs, they were rehydrated in PBS to the density of 5% (w/v), incubated at 37°C for 30 min. The rehydrated µRBs were then transferred into a 1 mL syringe, and injected through a 16 gauge needle to check if the µRBs can be ejected smoothly and homogeneously.

The in-situ crosslinking was then checked by adding 20 U/ml thrombin into the µRBs that were ejected into a custom-made cylinder mold. After incubating at 37°C for 20-30 minutes, the integrity of the formed scaffolds was examined.

To fabricate the cell-laden μRB scaffolds, the rehydrated µRBs were mixed with ASCs and injected through a 16 or 20 gauge needle into custom-made cylinder molds (50 mm^3^). The µRB density was varied at 5% (w/v) or 7.5% (w/v), and ASCs were encapsulated at a density of 20 million/ml. Cell/µRB mixture were crosslinked into macroporous cell-laden scaffolds by adding 5µl thrombin (20 U/ml). The ASC-laden μRB scaffolds were then transferred into 24-well plates for culture.

The ASC-laden µRB scaffolds without passing through injection were made as controls. Briefly, the rehydrated μRBs mixed with ASCs were sandwiched between two glass slides (with a gap of 0.5 mm), and thrombin was added subsequently to induce intercrosslinking. Samples were incubated at 37°C and for 24 h, then punched out into circular samples using biopsy puncher (3.5 mm in diameter) and transferred to 24-well plates for culture.

ASCs viability was tested by Live/Dead staining and BLI immediately after injection into 24 well plate. To test cell viability using BLI, D-luciferin (Caliper Life Sciences, Hopkinton, MA) was added to ASC-laden µRBs in 24 well plate to a final concentration of 150 μg/ml immediately after injection. Photon counts per second were recorded using an IVIS200 (Xenogen, Alameda, CA) imaging system and analyzed with Living Image 3.2 software (Caliper Life Sciences, Hopkinton, MA). Each well was scanned every 15 seconds until the peak signal was reached. Changes in bioluminescence intensity over time were measured and are presented as total flux values in photons/second for each well.

### BMP-2 loading and release from μRB-based scaffolds

To encapsulate BMP-2 into μRB-based scaffold, 5 mg μRBs were rehydrated in 100 μl BMP-2 solution (10 μg/ml). After that, 10 μl μRBs were injected and crosslinked in situ by thrombin, resulting in 10 scaffolds with 100 ng BMP-2 loaded per scaffold.

To assess the release of BMP-2 in vitro, scaffolds containing 100 ng BMP-2 were incubated in 24 well plates with serum containing medium. At each time point, the supernatant was collected, and 1ml fresh medium was added into the well. The amount of released BMP-2 was measured by using BMP-2 ELISA kit (Peprotech, Rocky Hill, NJ).

### Critical-size cranial defect surgery

Male FVB mice (7-week old, Charles River Laboratories, Hollister, CA) were used for cranial defect surgery. Briefly, mice were anesthetized with 2.5% isoflurane, and after removing the overlying pericranium, 3.3-mm cranial defects were created on the right parietal bone using a trephine drill without damaging the underlying dura mater. 10 µL of rehydrated µRBs was injected and filled into the defect, 1 µL of thrombin (200 U/mL) was added to allow in situ crosslinking. These groups are designated as “inject”. To assess the effect of injection, the scaffolds fabricated using the sandwich method without passing through injection were included and designated as “implant”. To assess the effect of BMP-2, the injected µRBs containing 100 ng BMP-2 per scaffold were included and designated as “inject+BMP-2”. All scaffolds were made as acellular and ASC-laden groups. The mice with defect but no treatment were included as negative control, designated as “non-treated”.

### Bioluminescence imaging (BLI)

To evaluate cell viability after transplantation into cranial defect mice, BLI was performed in cranial defect mice that received injected or implanted ASC-laden μRB-based scaffolds from day 0 to day 14. Mice received D-luciferin (150 mg/kg) by intraperitoneal injection were placed on a heated table (37°C) with nose cone and imaged using the IVIS Spectrum system (Caliper Life Sciences, Hopkinton, MA) under 2% isoflurane anesthesia at 30-s exposure time. Each mouse was scanned every 2 minutes until the peak signal was reached. Radiance was quantified in photons per second per centimeter squared per steradian.

### X-ray microtomography

To evaluate bone regeneration of cranial defect mice, every week from week 0 to week 8, the mineralization level of the scaffolds was monitored via X-ray microtomography using a large-field Inveon PET-CT (GE, Washington, D.C.), with settings of 80 kVp X-ray voltage, 500 lA anode current, 80 um voxel resolution, and 500 ms time for each 360 degree rotational step. The two-dimensional projection images were reconstructed into three-dimensional models with Microview (Parallax innovations Inc, Ilderton, Canads). Voxels at the supraoccipital part of the occipital bone were used as the threshold to identify mineralized bone formation. Percentage healing in each mouse was determined by calculating the percentage of reduction in the defect area using image J (NIH).

### Histology and immunostaining

Calvarial of mice were harvested at day 3, week 2 and week 8 for histology. Tissues were fixed overnight at 4% paraformaldehyde, demineralized for 2 weeks in 16% ethylenediaminetetraacetic acid, and embedded in OCT for cryo-sectioning. Tissue morphology was examined by H&E staining (Sigma, St. Louis, MO, USA) and Masson trichrome staining (Thermo Scientific, Waltham, MA) according to manufacturer's instructions. For immunostaining, 20μm sectioned slices were treated with blocking buffer consisting of 10% bovine serum albumin in 1X PBS and incubated with rat anti CD31 (BD Biosciences, San Jose, CA, USA, 1:50 dilution), rabbit anti luciferase (abcam, Cambridge, MA, USA, 1:50 dilution), mouse anti iNOS (BD Biosciences, 1:50 dilution) and mouse anti CD206 (Biolegend, San Diego, CA, USA, 1:50 dilution) overnight at 4 ℃. After washing in PBS for 3 times, sectioned slices were incubated for 1 h at room temperature with secondary antibodies. Nuclei was counterstained with Hoechst 33342 stain (Thermo Scientific) and images were taken under Zeiss fluorescence microscope. Sections were stained with all reagents without primary antibody for negative controls.

### Statistical analysis

All values were presented as mean ± standard deviation. Data were analyzed by analysis of variance, followed by Turkey post hoc comparisons. Two-tailed *p*<0.05 values were considered statistically significant. All statistical analyses were performed using GraphPad Prism (GraphPad Software, San Diego, CA).

## Results and Discussion

### Optimizing injectability of gelatin μRBs for forming in situ crosslinkable macroporous scaffolds

**Figure 1:**
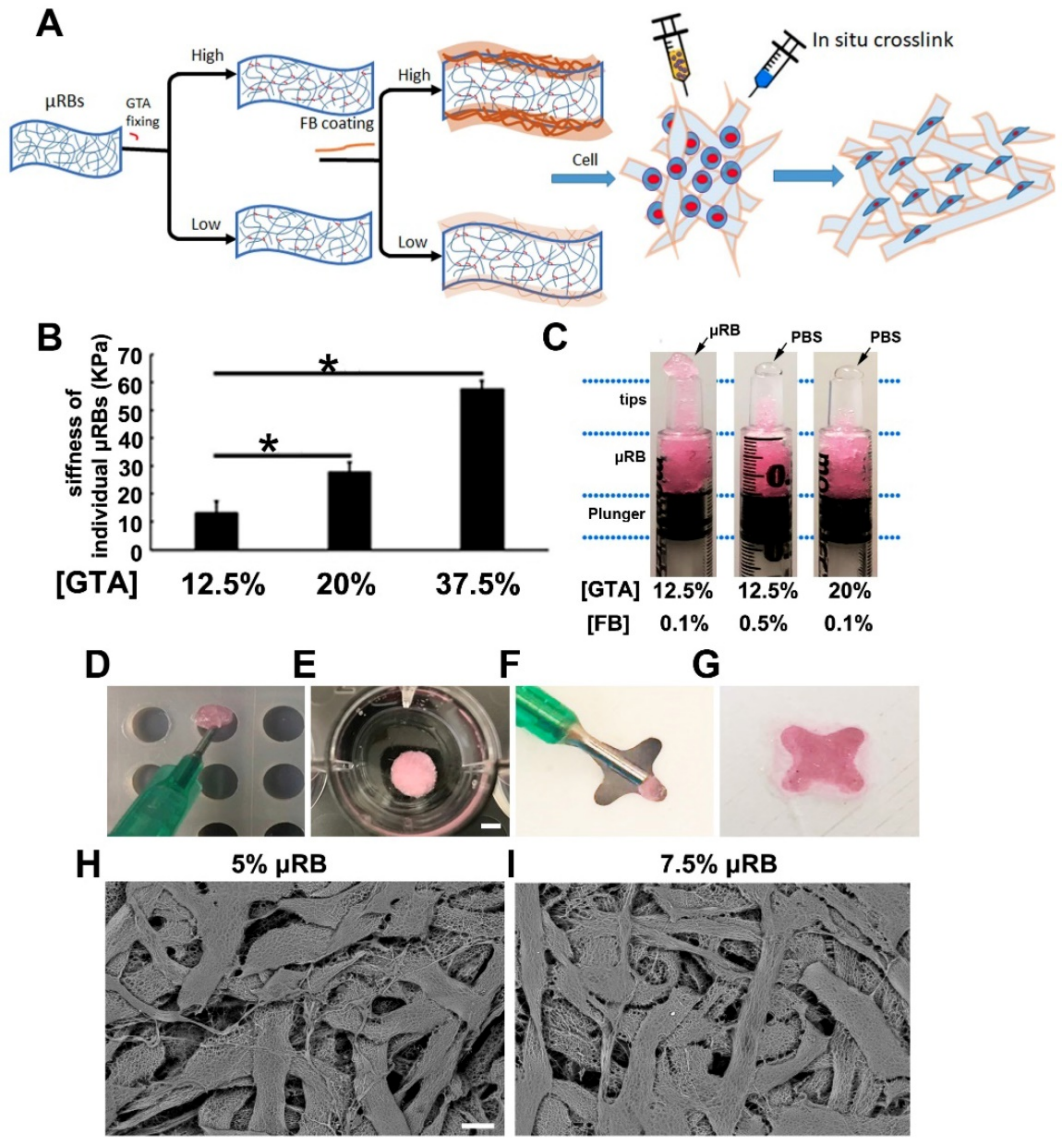
** Fabrication and characterization of μRB-based scaffold. (A).** Gelatin-based μRBs were firstly fabricated by wet spinning, then fixed with glutaraldehyde and coated with fibrinogen, which allow direct cell mixing and in situ crosslink with thrombin. **(B).** AFM measurement of surface stiffness of single μRB fixed with 12.5%, 20% and 37.5% GTA. *, *p*<0.05. **(C).** μRBs fixed with 20% GA or 0.5% fibrinogen squeezed PBS out during injection. Injection of optimized rhodamine-labeled μRB formulation into a cylinder mold **(D,** scale bar=5 mm**)** and 4-point star mold **(F)**, crosslinked with thrombin **(E and G)**. SEM imaging of 5% **(H,** scale bar=50 μm**)** and 7.5% **(I)** μRB-based scaffold.

Gelatin-based μRBs were fabricated by wet-spinning as we previously reported **(Figure 1A)**. The obtained gelatin-based μRBs were fixed using glutaraldehyde for 12 hours. According to our previous study, 12 h of glutaraldehyde incubation is required to obtain stable fixed µRB while shorter time glutaraldehyde treatment was insufficient to maintain the shape of μRB 28. To allow the in-situ formation of the macroporous scaffolds in vivo, we introduced the thrombin catalyzed crosslinking, which has been employed for fibrin glue, an FDA-approved biomaterial with demonstrated safety and biocompatibility 31. As compared to the previous used UV induced photo-crosslinking, this method could eliminate the potential cytotoxicity from the photo-initiator and limitation of light-penetration.

Unlike the injection of homogenous polymer precursor solution of most in-situ formation hydrogels, which can easily pass through the syringe and needles, the injection of the micron-sized µRB building blocks is subject to their stiffness and surface properties. As reported previously 28, the stiffness of individual μRB can be changed by the degree of glutaraldehyde (GTA) fixation, which was achieved by fixing µRBs in varying concentration of glutaraldehyde solution to consume different degrees of primary amines of µRBs. For example, increasing the degree of glutaraldehyde fixation from 12.5% to 37.5% led to a significant increased stiffness of µRBs, from 10 kPa to 60 kPa (Figure 1B). This increased stiffness made the µRBs more rigid and compromised the injectability. The µRBs fixed with 12.5% GTA can be injected smoothly through the syringe, but the ones fixed with 20% GTA was too rigid to be pushed through (Figure 1C). On the other hand, the fibrinogen coating which is introduced for in-situ crosslinking can also influence the injectability. Increasing fibrinogen concentration from 0.1% to 0.5% decreased the injectability. As shown in figure 1C, increasing both the degree of glutaraldehyde fixation from 12.5% to 20% and fibrinogen concentration from 0.1% to 0.5% led to phase separation (uRB phase and PBS phase) and make it unsuitable as an injectable cell carrier. This is probably due to increased stiffness and hydrophobicity of the μRBs. We found that the µRBs with 12.5% glutaraldehyde fixation and 0.1% fibrinogen coating was identified as the optimized injectable μRB formulation. The injectability allowed the µRBs to be filled into molds with different shapes (Figure 1D-F), can be in situ crosslinked by thrombin (Figure 1E-G), and demonstrate highly interconnected macroporosity (Figure 1H-I).

### Determine optimal μRB formulation that support high cell viability post-injection

**Figure 2:**
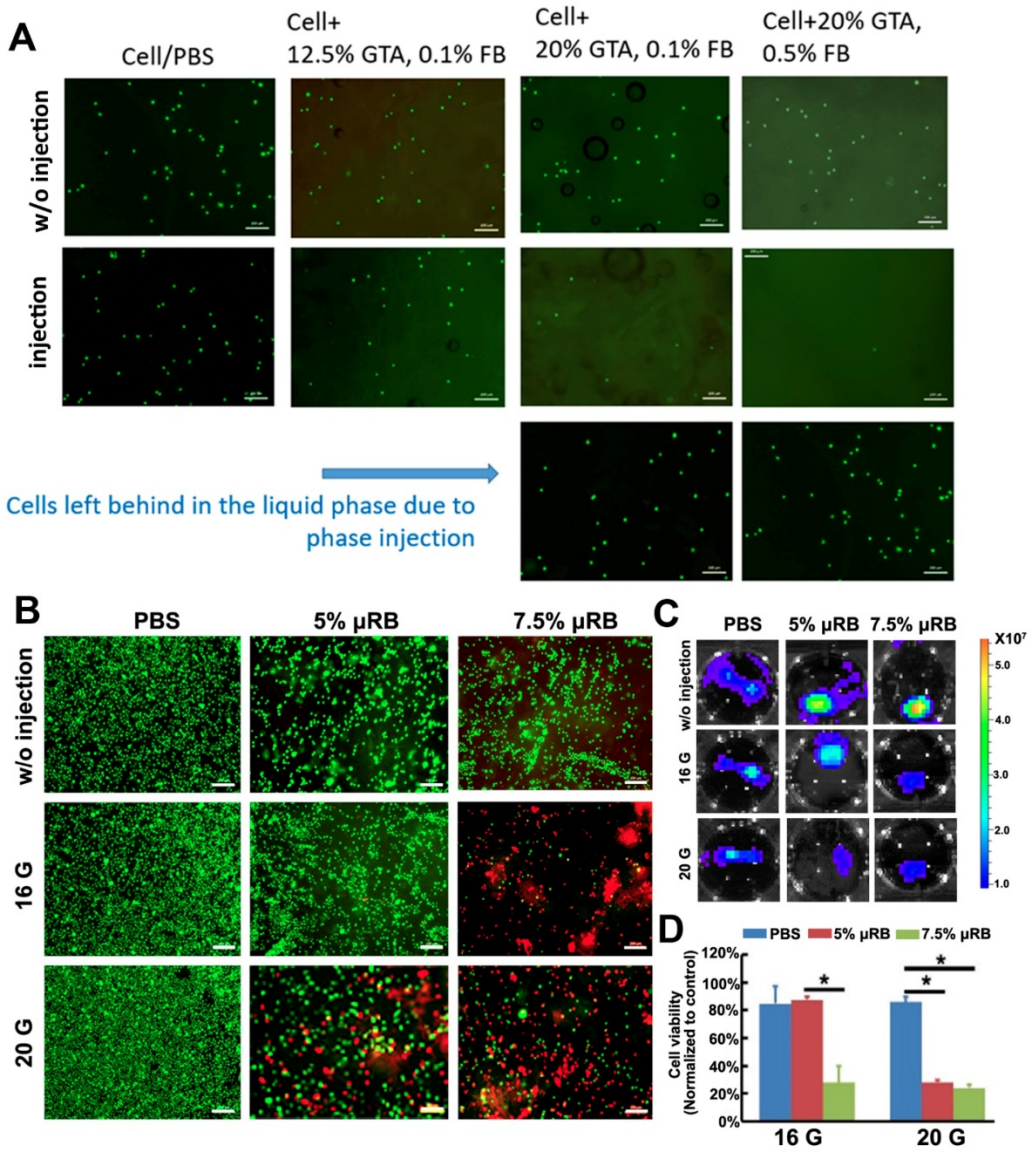
** Optimization of injectable μRB-based scaffold that support cell survival. (A).** Live dead staining of ASC encapsulated in different formulations of μRBs with and without injection**.** Green: live cells; Red: dead cells. Bar=200 μm. **(B).** Live and dead imaging of ASCs that encapsulated with 5% or 7.5% μRBs and injected through 16 gauge or 20 gauge syringe needle. Bar=200 μm. **(C).** Bioluminescence imaging of ASCs that encapsulated in 5% and 7.5% μRBs after injection through 16 gauge and 20 gauge needles. **(D).** Quantification data from (B). All data are presented as mean±S.D. *, *p*<0.05.

We then explored the injectability of different formulations of μRBs encapsulated with ASCs. We found that all formulations of μRBs supported high cell viability after encapsulation (Figure 2A). However, increasing GTA fixation from 12.5% to 20% and fibrinogen concentration from 0.1% to 0.5% led to fewer cells after injection, and the majority of cells/PBS were phase separated from μRBs and failed to be encapsulated after injection. This is because increasing GTA concentration or fibrinogen concentration both led to increased hydrophobicity of the μRBs, resulting in phase separation from cell/PBS mixture during injection. We identified the lead formulation to be μRBs fixed with 12.5% GTA, and coated with 0.1% fibrinogen, which did not cause phase separation and supported efficient cell encapsulation in μRB hydrogel post-injection (Figure 2A). One challenge associated with injectable matrices for cell delivery is the shear force-induced cell death during the injection 32, 33. We further determined the effects of varying µRB density (5% or 7.5%) and needle size (16 or 20 gauge) on cell survival. ASCs cell viability was examined by live and dead staining and bioluminescent imaging. Increasing the µRB density and decreasing the needle size can both increase the shear force sensed by the cells during injection. Indeed, we observed decreased the cell survival post-injection with higher µRB density and smaller needle size, probably due to the high shearing force (Figure 2B-C). Importantly, ASCs maintained their osteogenic capability post-injection, as confirmed by ALP and ARS staining (Figure S2, Supporting Information). Based on the cell viability results, we chose 5% μRBs fixed with 12.5% glutaraldehyde and coated with 0.1% fibrinogen as the optimal injectable formulation for the following in vivo studies. In the event where needles smaller than 16G are preferred for delivery, several strategies could be explored to further improve the cell viability after injection including decreasing the degree of GTA fixation or fibrinogen concentration, or decreasing μRB density.

### Injectable μRB-based scaffold support cell survival in vivo, and incorporation of BMP-2 further enhances cell proliferation

Conventional injectable hydrogels are usually nanoporous, which restricts cell spreading, growth, and tissue formation. To overcome these challenges, recent studies showed nanoporous hydrogels with dynamic cell-adaptable network can facilitate cell spreading and MSC osteogenesis. Feng et al reported a facile supramolecular gelatin hydrogels crosslinked by weak host-guest interactions that facilitate endogenous cell infiltration and migration 34. Recently highly dynamic network of cell-infiltratable and injectable gelatin hydrogel has also been reported for promoting bone regeneration by enhancing endogenous cell infiltration 35. The approach reported in our study is different from these previous studies in that the resulting scaffold is macroporous. Our µRBs serve as building blocks, which can be injected through syringe and form a macroporous scaffold in situ. As such, we could combine the injectability and macroporosity into the scaffolds for cell delivery and cell-based tissue regeneration.

**Figure 3:**
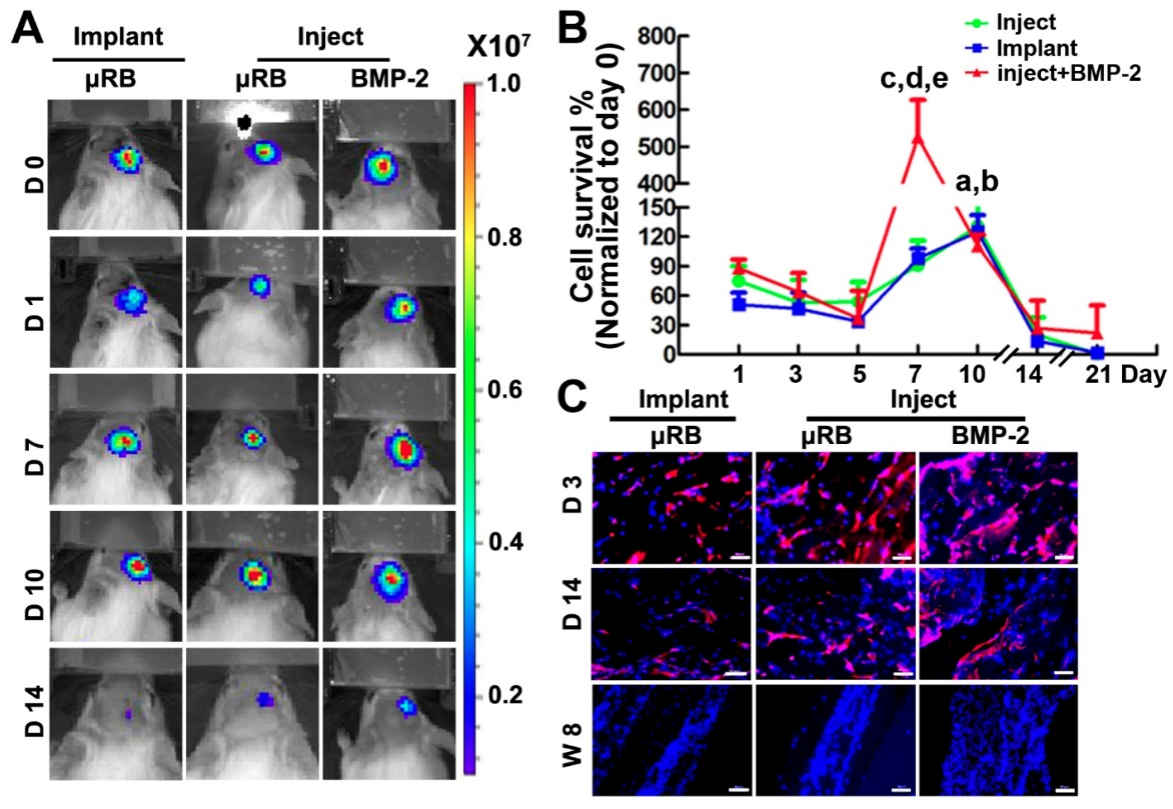
** Cell viability after transplantation into a mouse critical size cranial defect model. (A).** BLI of mice that implanted with ASC-laden μRB scaffold or injected with ASC-laden μRB scaffold (with and without BMP-2 incorporation) across different time points.** (B).** Quantitative data from (A). a, *p*<0.001, Day 10 vs Day 1 in mice treated with implanted μRBs; b, *p*<0.05, Day 10 vs Day 1 in mice treated with injected μRBs; c, *p*<0.001, Day 7 vs Day 1 in mice treated with injected μRBs+BMP-2; d, *p*<0.001, mice treated with injected μRBs+BMP-2 vs mice treated with injected μRBs; e,* p*<0.001, mice treated with injected μRBs+BMP-2 vs mice treated with implanted μRBs; All data are presented as mean±S.D. N=5 per group. **(C).** Immunostaining of luciferase in cranial defect mice implanted with ASC-laden μRB scaffold or injected with ASC-laden μRB scaffold (with and without BMP-2) at day 3, 7 and 14. Bar=50 μm.

By using BLI and immunohistochemical staining, we found that scaffolds supported more than 50% of ASCs survival one day after injection, and injectable µRB group showed slightly higher cell viability even compared to the implanted prefabricated group (positive control). In addition, both injectable and implanted µRB scaffolds supported cell proliferation, with BLI signal peaking at day 10 (131±34% in injection group and 125±17% in implantation group vs. day 0, *p*<0.05), highlighting the advantage of macroporosity on cell survival and proliferation. At day 10, cell number started to decline and minimum BLI signal could be detected from both groups after day 21 (Figure 3A-B). The drop of BLI signal indicates decreased number of viable cells, which may be due to nutrients competition between ASCs and infiltrated cells, such as fibroblasts and macrophages. It is also possible that increase amounts of infiltrated cells led to the formation of a dense fibrous tissue, which limits oxygen exchange and induces hypoxia, further increasing cell death 36. Incorporation of BMP-2 in the µRB scaffolds significantly increased cell proliferation, with a 5-fold increase of BLI signal peaking at day 7, compared to the 1.2-fold increase in the group without BMP-2 (Figure 3A-B), indicating in situ BMP-2 delivery is beneficial for ASCs proliferation. To further confirm the cell retention and distribution, immunostaining of luciferase was performed in all the three groups. The number of transplanted ASCs (luciferase positive cells) increased from day 3 to day 7, and substantially decreased from day 7 to day 14, which was consistent with BLI data (Figure 3C).

### In vivo biodegradation of μRB scaffolds in cranial defects

**Figure 4:**
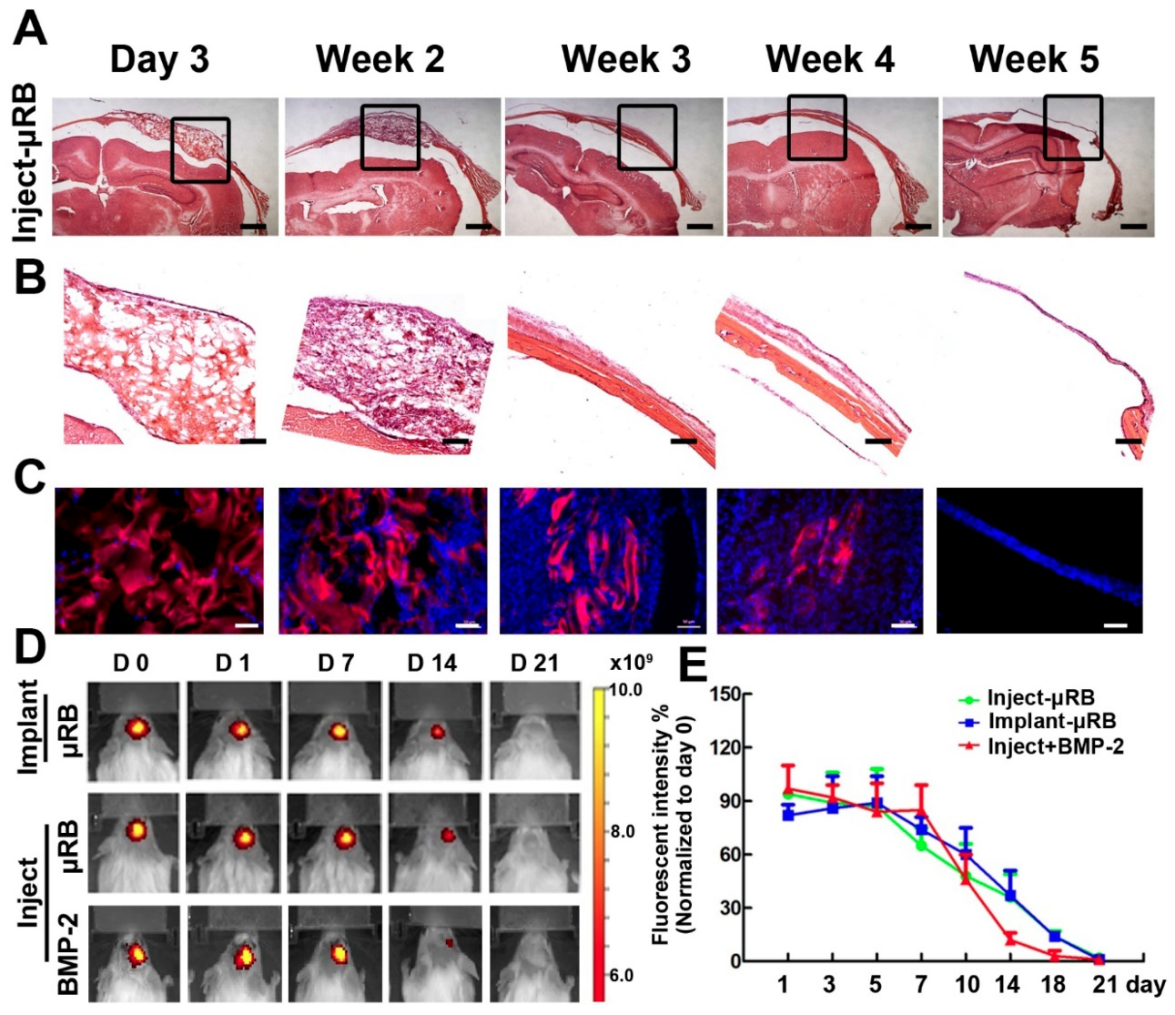
** Degradation of μRB-based scaffolds in a mouse critical size cranial defect model. (A).** H&E staining of injected μRB-based scaffolds harvested from cranial defect mice at day 3, week 2, week 3, week 4 and week 5. **(B).** High magnification of the inserts of (A). **(C-D).** Fluorescence imaging of injected Alex flour 700-labeled μRB scaffolds harvested from cranial defect mice at various time points. Bar=50 μm. **(E).** Quantitative data from (D). All data are presented as mean±S.D. N=5 per group.

To investigate biodegradation of μRB scaffold in vivo, μRBs were labelled with Alex flour 700 dye and injected into cranial defects. H&E staining (Figure 4A-B) and fluorescence imaging (Figure 4C-E) results showed that μRB scaffold maintained its macroporosity for 2 weeks in vivo. A substantial decrease in scaffold size was observed at week 3, suggesting substantial degradation of the µRB scaffolds. By week 5, minimum µRB scaffolds could be identified from either H&E or fluorescent images. Neither addition of ASC nor BMP-2 affect the degradation of μRB based hydrogel. Two mechanisms including hydrolysis and enzymatic degradation are responsible for gelatin-based hydrogels degradation. The main composition of gelatin after degradation contains 19 amino acids, predominantly glycine, proline and hydroxyproline. Gelatin degradation takes place in two sequential steps. In the first step, gelatinases degrade gelatin into polypeptides. Then, the polypeptides are further degraded into amino acids. Previous studies show that composition of gelatin after degradation are highly biocompatible 37. In our study, we did not find adverse inflammatory tissue reaction in vivo after injection of μRB based hydrogels (Figure 6).

### Injectable gelatin μRB-based scaffold containing BMP2 synergize with ASCs to accelerate mineralized bone formation in vivo

**Figure 5:**
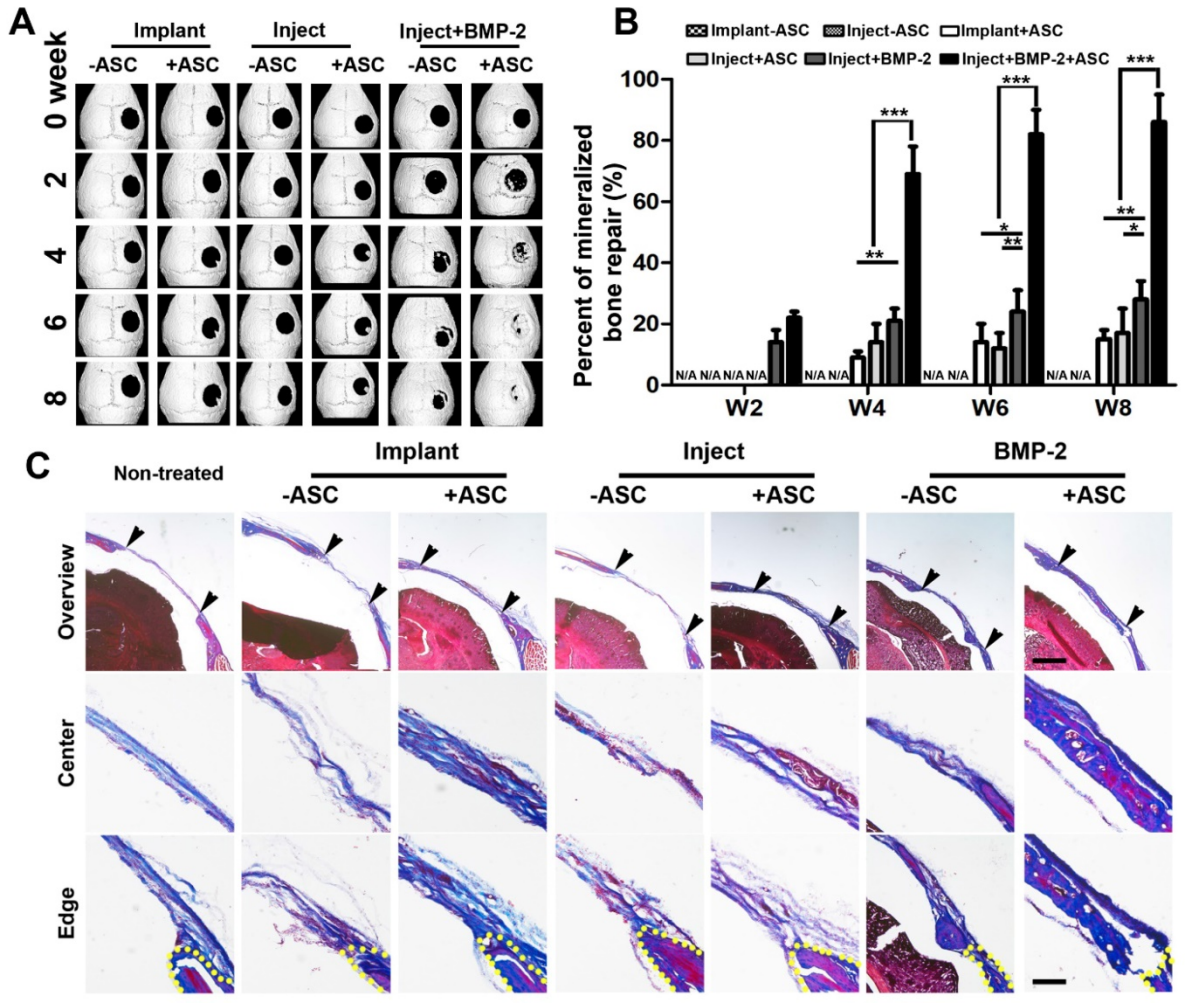
** Micro-CT imaging of cranial defect mice across different time points and histological cross sections of mouse cranial defects at week 8. (A).** Representative micro-CT images of cranial defect mice treated with injected and implanted μRB scaffolds, and BMP-2 incorporated μRB scaffolds, with or without ASCs. **(B).** Quantification data from (A). Percent of bone healing was normalized to the defect size at week 0. *, *p*<0.05; **, *p*<0.01; ***, *p*<0.001. Data are presented as mean±S.D. N=5 per group. **(C).** Trichrome staining of the defect center and edge of cranial defect mice treated with injected and implanted μRB scaffolds, and BMP-2 incorporated μRB scaffolds, with or without ASCs, Bar=1mm (first row) and 200 μm (middle and last row).

We then evaluated the efficacy of injectable gelatin µRB scaffolds for supporting bone regeneration in vivo using an immunocompetent mouse critical-size cranial defect model. Mineralized bone formation was monitored for up to 8 weeks by X-ray microtomography (micro-CT). Both injectable and implantable µRB scaffolds containing ASCs led to comparable degree of bone repair, though still slow with only ~14% bone defects filled by week 8 (Figure 5). To further accelerate mineralized bone formation, BMP-2 was directly coated onto μRBs, and a sustained release of BMP-2 from the µRB scaffolds was obtained without additional modification (Fig. S3, Supporting Information). This could be due to the physical adsorption mediated through binding with fibrinogen coating 38, 39. Bone morphogenetic proteins (BMPs) are responsible for bone formation during embryogenesis and bone regeneration and remodeling. The osteoinductive action of BMPs, especially BMP-2, has led to wide use for effective bone regeneration 40, 41. Our results showed that BMP-2 significantly accelerated bone regeneration even without ASCs, filling up to 28%±6% bone defects by week 8. Impressively, Co-delivery of ASCs with 100 ng BMP-2 in the µRB scaffolds exhibited great synergy, substantially accelerating bone regeneration to 86%±9% by week 8 (Figure 5A-B), Consistent with imaging, Masson's trichrome staining also demonstrated extensive collagen-rich tissue ingrowth in bone defects using injectable µRB scaffolds containing BMP-2, and group containing both ASCs and BMP-2 leading to the most intense collagen deposition (Figure 5C). Groups without ASCs generally showed less collagen deposition, which was more similar as non-treated control group.

### Assessing inflammatory response of μRB scaffold in vivo

Emerging evidence has highlighted the importance of inflammatory response in multiple stages of bone formation 42. In the acute stage (0-7 days after bone defect), macrophages derived from a distinct population of blood monocytes are recruited and rapidly infiltrated into the defect area, and the majority are M1 type inflammatory macrophages 43. In the subacute phase (7-28 days), inflammatory M1 type macrophages gradually shift to anti-inflammatory M2 type, which has been reported to be associated with bone regeneration 44, 45. And the later chronic phase is characterized by few macrophages or giant cells.

**Figure 6:**
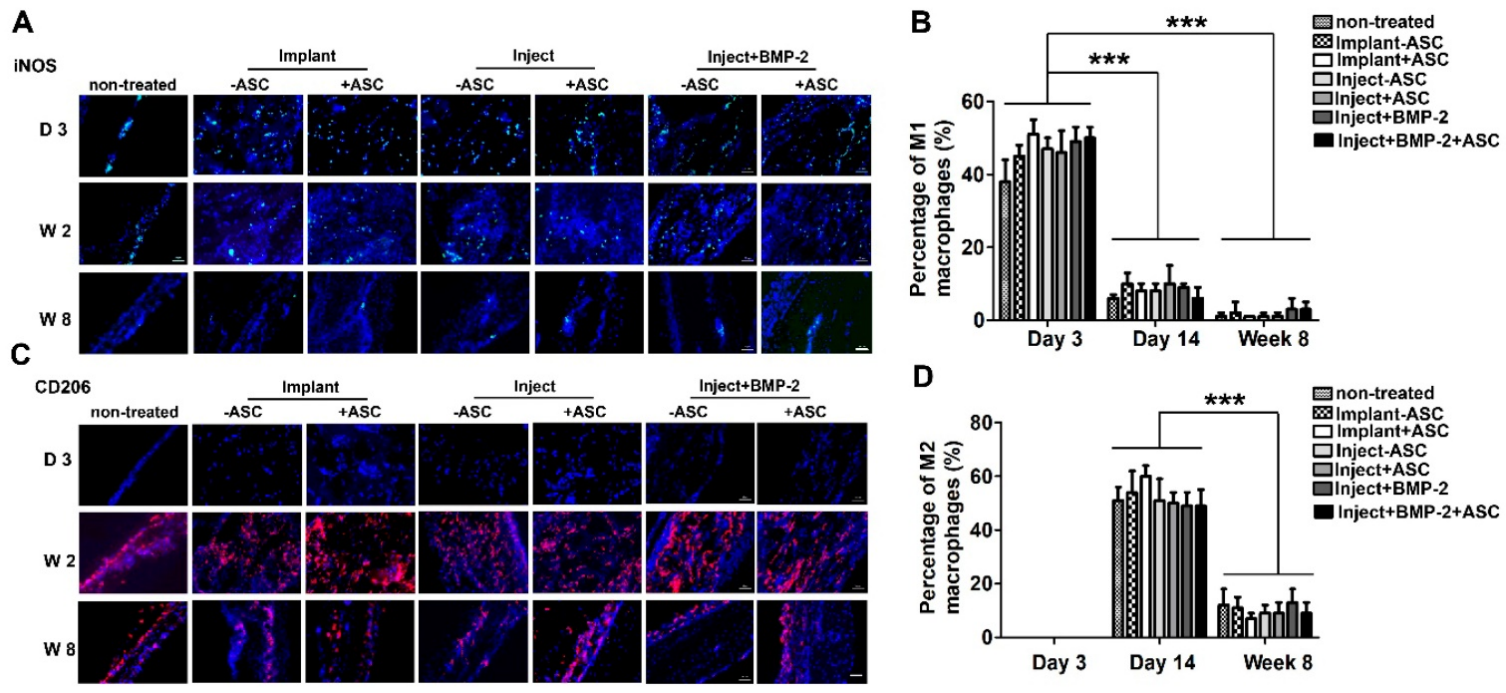
** Inflammatory response of μRB scaffolds in a mouse critical size cranial defect model.** Immunostaining of M1 type macrophage marker iNOS **(A)** and M2 type of macrophage marker CD206 **(C)** in non-treated mice, mice transplanted with implanted ASC-laden μRB scaffold, injected ASC-laden μRB scaffold (with and without BMP-2 incorporation) and acellular μRB scaffold at day 3, day 14 and week 8. (**B**). Quantitative data from (A). ***, *p*<0.001. (**D**). Quantitative data from (C). ***, *p*<0.001. Bar=50 μm. All Data are presented as mean±S.D. N=5 per group.

In this study, we have specifically characterized the inflammatory response by immunostaining of specific markers of M1 and M2 type macrophages at early (day 3), intermediate (week 2) and later stage (week 8). M1 and M2 macrophages were identified by immunofluorescent staining of M1 and M2 markers iNOS and CD206, respectively (Figure 6). Untreated cranial defect was included as a control to assess the inflammation caused by the surgery itself. The pro-inflammatory M1 type macrophages were identified in all groups at early time point (day 3), indicating acute inflammation caused by the cranial defect surgery. An inflammatory response with kinetic of macrophage population desirable for bone formation was observed in all of our experimental groups (Figure 6). Specifically, the number of inflammatory M1 macrophages dramatically decreased from day 3 to day 14 and few was detected at week 8, whereas the anti-inflammatory M2 type macrophages showed an opposite trend, with highest M2 cell number found at day 14. The shift of M1 type to M2 type is desirable for bone formation in vivo. Importantly, groups treated with injectable or implantable gelatin µRB scaffolds showed comparable percentage of macrophage as the untreated defect (control). In addition, we noticed that there is no significant difference of inflammatory response between cellular and acellular groups, suggesting ASCs do not affect macrophage polarization in immunocompetent mouse cranial defect model.

### Vascularization of μRB scaffold in vivo

**Figure 7:**
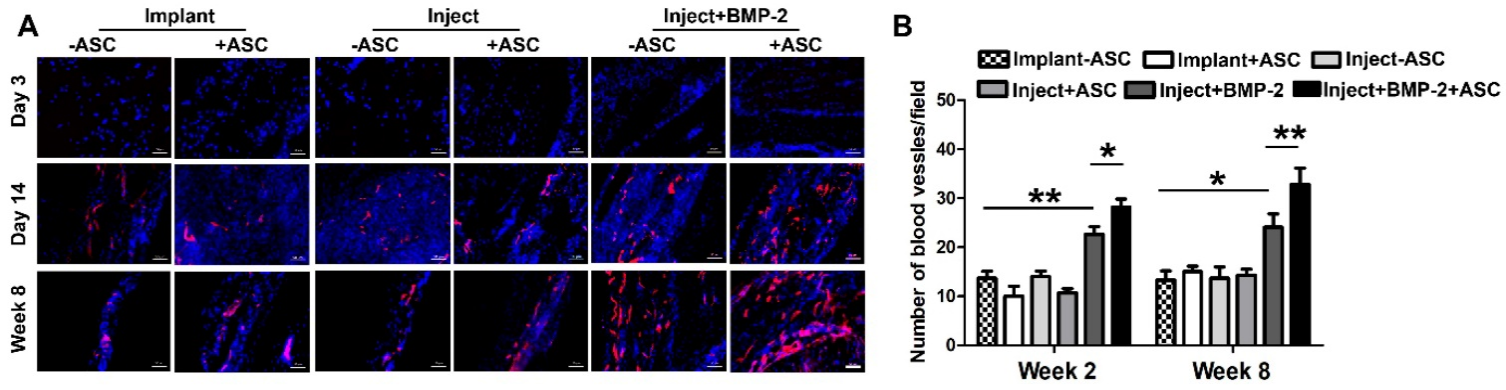
** Vascularization in cranial defects treated with implanted and injected μRB scaffolds. (A).** Immunostaining of endothelial cell marker CD31 in cranial defect mice transplanted with implanted and injected ASC-laden μRB (with and without BMP-2) scaffold and acellular μRB-based scaffold at day 3, 14 and week 8. Red=CD31, Blue=DAPI. Bar=50 μm. **(B).** Quantitative data from (A). *, *p*<0.05; **, *p*<0.01. All Data are presented as mean±S.D. N=5 per group.

Bone is a highly vascularized biomineralized connective tissue, and vascularization of large bone grafts is one of the main challenges of bone tissue engineering 46. Lack of vasculature results in ineffective integration of grafts to the host vasculature upon implantation 47, 48. Use of macroporous materials that contain interconnected channels could promote the survival of seeded cells following transplantation due to enhanced rapid nutrient diffusion 49. We next assessed the vascularization in the µRB scaffolds post-transplantation in vivo, which is also critical for bone healing. Here, immunostaining of endothelial cell marker-CD31 was performed. Endothelial cell was not observed in μRB scaffold at day 3, but emerged from day 14 and are present up to week 8 (Figure 7A), suggesting vascularization in μRB scaffolds. Incorporation of BMP-2 significantly enhanced the vascular density, which synergize with ASCs to induce the highest degree of vascular density by week 8 (Figure 7B). This is in consistence with some previous studies, which have well documented the ability of BMP-2 for promoting angiogenesis through multiple pathways including P38, ERK and Akt/m-TOR in vitro 50, 51. This enhanced vascularization by the BMP-2 is also correlated with the enhanced ASC proliferation (Figure 3) and bone formation in vivo (Figure 5), which could be due to the enhanced nutrient supply as a result of the vasculature.

## Conclusion

In summary, here we fabricated a gelatin-based µRB scaffolds for cell delivery, which is specifically optimized to be injectable and in situ crosslinkable. The µRBs are crosslinked by fibrinogen mediated by a blood clotting mechanism, which is biocompatible and widely used in fibrin glue as bio-adhesives. The injectability of the µRBs was optimized by adjusting the degree of glutaraldehyde fixation, fibrinogen coating, μRB density and syringe needle size. The optimized µRBs well supported cell survival and proliferation in vivo post-injection. Using an immunocompetent mouse critical-size cranial defect model, we further demonstrated that injected μRB-based scaffold supported ASC based bone formation in vivo, which performed comparably to implanted μRB-based scaffold. In addition, the µRBs can be used for co-delivery of BMP-2 with ASCs, which synergistically enhanced bone regeneration in vivo and enhanced vascularization. Our injectable μRB-based scaffold allows easy delivery of stem cells and growth factors into the defect area in a minimal invasive manner, and can be broadly used as injectable macroporous matrices to deliver various cell types for tissue regeneration.

## Supplementary Material

Supplementary figures and tables.

## Abbreviations

ASC: adipose-derived stromal cell
ALP: alkaline phosphatase
ARS: alizarin red
BMP-2: Bone morphogenetic protein 2
µRB: microribbon
