# Supplementary material for: Injectable and in situ crosslinkable gelatin microribbon hydrogels for stem cell delivery and bone regeneration in vivo
Source: Theranostics. 2020 May 15;10(13):6035–47. doi: 10.7150/thno.41096 (PMC7254995; doi:10.7150/thno.41096)
Supplement: Supplementary file 1 — Supplementary figures and tables. [file thnov10p6035s1.pdf]

Supporting Information

Injectable and in situ Crosslinkable Gelatin Microribbon Hydrogels for Stem Cell Delivery and Bone Regeneration in vivo

Yaohui Tang[1], Xinming Tong[1], Bogdan Conrad[2], Fan Yang[1,3]*

Identification of ASCs

Flow cytometry was performed to characterize ASCs, and 85.5% of these cells were CD90 positive, 44.2% and 17.9% of the cells were CD105 and CD73 positive, and less than 5% were CD45 positive, (Figure S1A-D), and they were capable of undergoing osteogenic and adipogenic differentiation when cultured in osteogenic and adipogenic medium, as demonstrated by ALP, ARS and oil red staining (Figure S1E-G).

Characterization of osteogenic differentiation

Osteogenic differentiation of ASCs encapsulated in 5% μRBs after injection through 16 gauge needles (Inject group) or pre-fabricated μRBs without injection (Implant group) were characterized by ALP staining at day 7 and ARS staining at day 21 (Figure S2).

Characterization of BMP-2 release in vitro

ELISA test was used to quantify BMP-2 release in vitro. As shown in supplementary figure 3, a weak burst release within the first day was observed and a

sustained release of the incorporated BMP-2 was detected in the following several weeks. 40% BMP-2 was released in total until day 14.

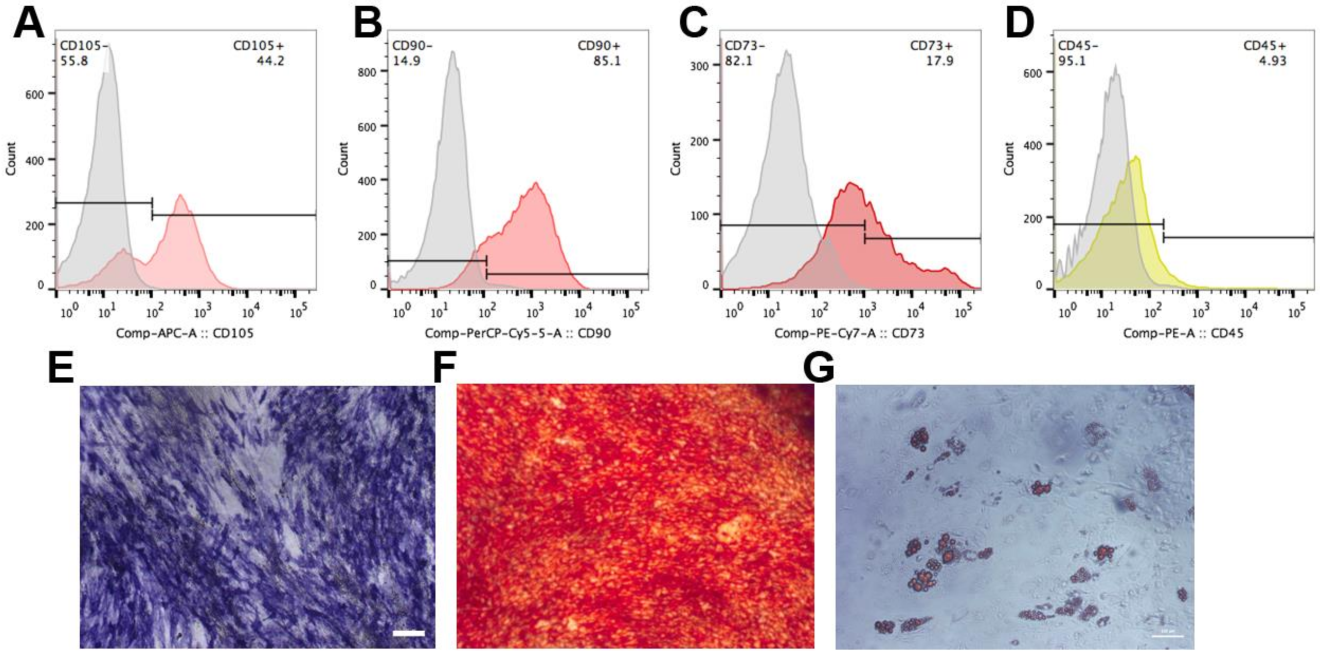

Figure S1. Characterization of mouse adipose-derived stem cells (ASCs). Flow cytometry was performed for CD105 (A), CD90 (B), CD73(C), and CD45 (D). ALP (E), ARS (F) and Oil red (G) staining of ASCs that cultured in osteogenic differentiation medium and adipogenic differentiation medium. Scale bar: 100 μm.

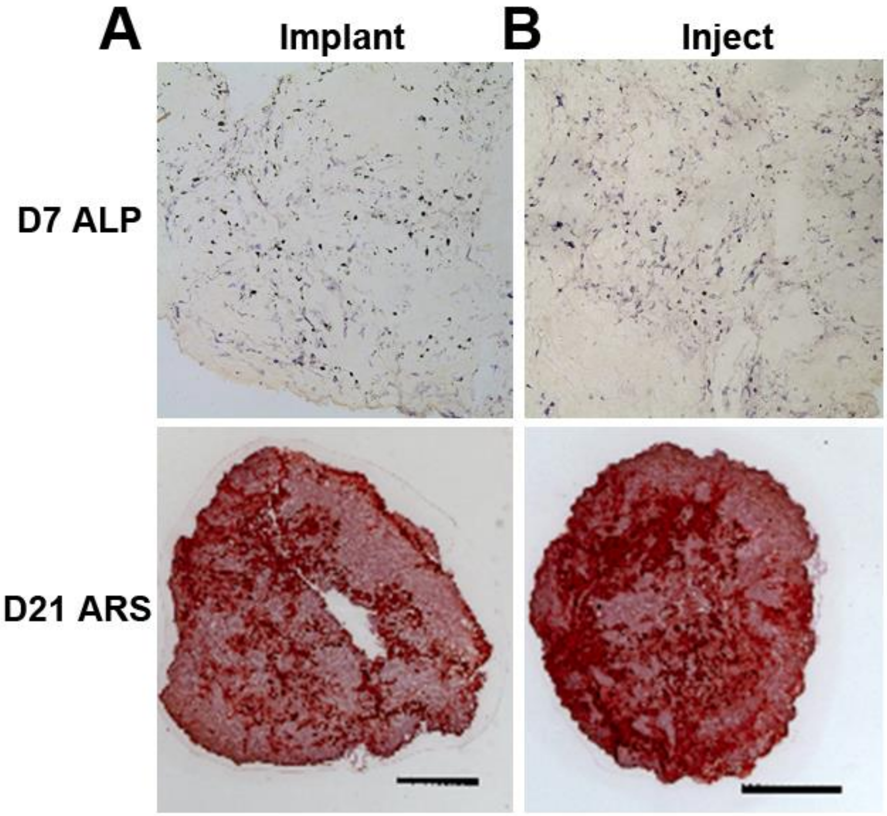

Figure S2. Osteogenic differentiation of ASCs encapsulated in μRB-based scaffold. ALP staining at day 7 and ARS staining at day 21 of ASCs that encapsulated in prefabricated μRB-based scaffold without injection (A) and μRB-based scaffold post-injection (B). Bar=2 mm.

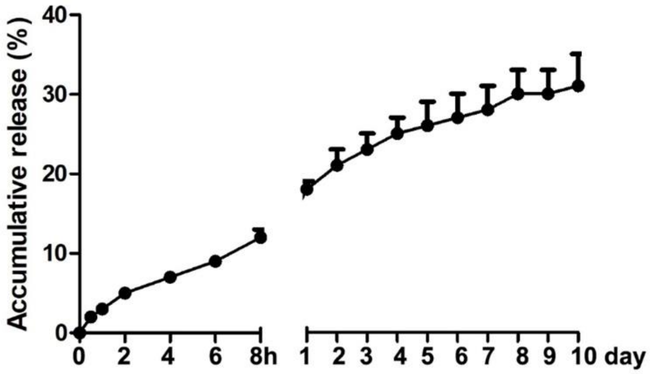

Figure S3. Characterization of BMP-2 release. Cumulative BMP-2 release from μRB scaffolds. Data are presented as mean±S.D. N=3 per group.
